# Simultaneous Spectrophotometric Determination of Drotaverine Hydrochloride and Paracetamol in Tablet

**DOI:** 10.4103/0250-474X.62241

**Published:** 2010

**Authors:** Sonali Mahaparale, R. S. Telekone, R. P. Raut, S. S. Damle, P. V. Kasture

**Affiliations:** Dr. D. Y. Patil College of Pharmacy, Akurdi, Pune-411 044, India

**Keywords:** Drotaverine hydrochloride, paracetamol, Q analysis and first order derivative method, ultraviolet spectrophotometry

## Abstract

Two simple, accurate and reproducible spectrophotometric methods; Q analysis and first order derivative method have been described for the simultaneous estimation of drotaverine hydrochloride and paracetamol in combined tablet dosage form. Absorption maxima of drotaverine hydrochloride and paracetamol in distilled water were found to be 303.5 nm and 243.5 nm respectively. Beer's law was obeyed in the concentration range 5-50 μg/ml for drotaverine and 5-60 μg/ml for paracetamol. In Q analysis method, two wavelengths were selected at isobestic point (277 nm) and λ_max_ of paracetamol (243.5 nm). In first order derivative method, zero crossing point for drotaverine hydrochloride and paracetamol were selected at 303.5 nm and 243.5 nm, respectively. The results of two methods were validated statistically and recovery studies were found to be satisfactory.

Drotaverine hydrochloride (DRO) and paracetamol (PAR) are available in tablet dosage form in the ratio of 2:12.5. Chemically, drotaverine hydrochloride is 1- [(3,4-diethoxy phenyl) methylene]-6,7-diethoxy-1,2,3,4-tetrahydroisoquinoline[1]. It is an analog of papaver and is used mainly as an antispasmodic, smooth muscle relaxant[2]. DRO is official in Martindale Pharmacopoeia[2]. Literature survey reveals that UV spectrophotometry[3,4] and HPLC[5,6] methods are reported for determination of DRO. Paracetamol is 4-hydroxy acetanilide[1], has analgesic and antipyretic activity[1,2]. PAR is official in Martindale Pharmacopoeia[2], IP[7], BP[8] and USP[9]. Literature survey revealed that UV spectrophotometry[7,8] and HPLC[7,9] methods are reported for determination of PAR from its pharmaceutical formulations. There are no reported UV spectrophotometric methods for simultaneous estimation of both drugs in combined dosage form. Hence an attempt has been made to develop simple, sensitive, rapid, accurate, precise and economical UV spectrophotometric methods for the simultaneous estimation of DRO and PAR in bulk and tablet dosage forms.

Standard gift samples of drotaverine hydrochloride and paracetamol were procured from Vishnu Chemicals Ltd., Hyderabad. Combined drotaverine hydrochloride and paracetamol tablets (Label claim 80 mg DRO and 500 mg PAR) were purchased from local market. A Shimadzu UV/Vis double beam spectrophotometer, model 1700, with matched quartz cells corresponding to 1 cm pathlength and spectral bandwidth of 2 nm was used for performing the analysis. Methanol AR grade and distilled water were used as solvents in the study.

The stock solutions (100 μg/ml) of DRO and PAR were prepared separately by dissolving accurately about 10 mg of each drug in 25 ml methanol (AR grade) in 100 ml volumetric flasks and making up the volume with distilled water. Working standard solutions of DRO and PAR were prepared separately from standard stock solution. These solutions were scanned in the spectrum mode from 400.0 to 200.0 nm. The maximum absorbance of DRO and PAR was observed at 303.5 and 243.5 nm, respectively. The linearity of DRO and PAR was found to be in the concentration ranges of 5-50 μg/ml and 5-60 μg/ml, respectively, at their respective maximas. The coefficients of correlation were found to be 0.9992 for DRO and 0.9995 for PAR.

In the Q analysis method, absorbances were measured at two wavelengths, one being the isoabsorptive point and other being the wavelength of maximum absorption of one of the two components. From overlain spectra of DRO and PAR (fig. 1), absorbances were measured at the selected wavelengths, 277.0 nm (isoabsorptive point) and 243.5 nm (λmax of PAR). Appropriate dilutions were made to obtain mixed standard solutions in Beer–Lamberts' range for DRO and PAR in the ratio of 2:12.5 from 4, 8 μg/ml of DRO and 25, 50 μg/ml of PAR, respectively and scanned in the spectrum mode from 400.0 to 200.0 nm. The absorbances of mixed standards were measured at selected wavelengths. The concentration of each component present in mixed standard was calculated by mathematical treatment of the following mentioned equations. For drotaverine, C_1_ = (Q_m_–Q_y_)/(Q_x_–Q_y_)×A_1_/a_1_, for paracetamol, C_2_=(Q_m_–Q_x_)/(Q_y_–Q_x_)×A_1_/a_2_, where C_1_ is concentration of DRO, C_2_ is concentration of PAR, A_1_ is absorbance of sample at isoabsorptive wavelength 277.0 nm and a_1_ (0.0142), a_2_ (0.0143) are absorptivities of DRO and PAR at isoabsorptive wavelength 277.0 nm, respectively. Q_x_ is the absorptivity of DRO at 243.5 nm/absorptivity of DRO at 277.0 nm, Q_y_ is the ratio of absorptivity of PAR at 243.5 nm/absorptivity of PAR at 277.0 nm and Q_m_ is the ratio of absorptivity of sample solution at 243.5 nm/absorptivity of sample solution at 277.0 nm.

**Fig. 1:**
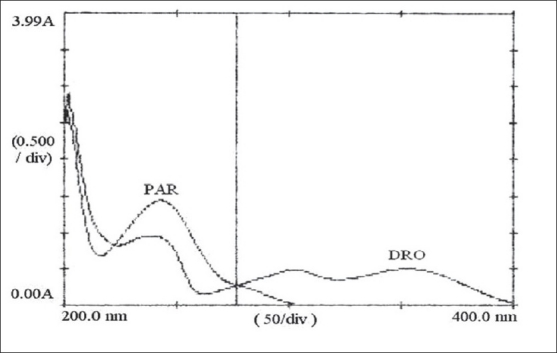
Overlain spectra of drotaverine hydrochloride and paracetamol

In the first order derivative method, solutions of 10 μg/ml of DRO and PAR were prepared separately. Both the solutions were scanned in the spectrum mode from 400.0 to 200.0 nm. The absorption spectra thus obtained were derivatized from first to fourth order. First order derivative (n=1) was selected for analysis of both the drugs. The zero crossing wavelengths, 303.5 nm and 243.5 nm were selected for DRO (fig. 2) and PAR (fig. 3), respectively. Working standard solutions of DRO and PAR were prepared separately and scanned in the spectrum mode from 400.0 to 200.0 nm. The absorption spectra obtained were derivatized to obtain first order derivative spectra. The absorbances of DRO and PAR were measured at 243.5 and 303.5 nm, respectively and working calibration curves of both the drugs were plotted separately. The coefficients of correlation were found to be 0.9994 for DRO and 0.9992 for PAR. The concentration of individual drug present in the mixture was determined against calibration curve in quantitation mode.

**Fig. 2:**
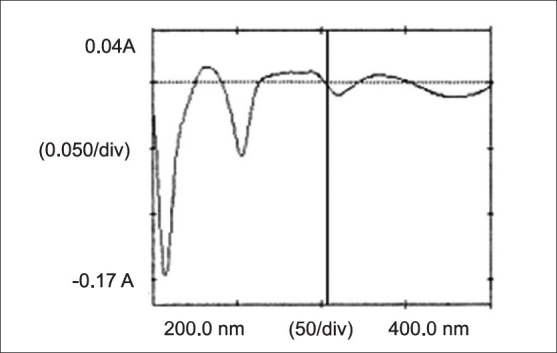
First order derivative spectra of drotaverine hydrochloride

**Fig. 3:**
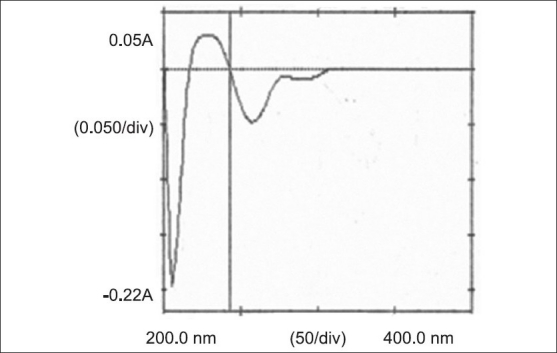
First order derivative spectra of paracetamol

For determination of both drugs from commercial formulations, twenty tablets were weighed and crushed to obtain fine powder. The powder sample equivalent to 4 mg of DRO and 25 mg of PAR was weighed and transferred to 100 ml volumetric flask. The drug content was dissolved in 25 ml of methanol AR grade, and was kept in ultrasonicator for 30 min. Finally, the volume was made up to the mark with distilled water. The solution was filtered through Whatmann filter paper No. 41. The filtrate was further diluted to obtain mixed sample solutions in Beer–Lamberts' range for each drug in the ratio of 2:12.5 from 4 and 8 μg/ml of DRO and 25 and 50 μg/ml of PAR, respectively. For the Q analysis method, the absorbances of mixed sample solutions were measured at 243.5.0 and 277.0 nm, respectively. The concentration of each drug in sample was calculated using above mentioned equations given in Q analysis method.

For the first order derivative method, the absorbances of mixed sample solutions were measured at 243.5 and 303.5 nm. The concentrations of DRO and PAR present in the sample solution were determined against calibration curve in quantitation mode. The tablet analysis results obtained by proposed methods were validated by statistical evaluation[10,11] (Table 1). To study accuracy and reproducibility of proposed methods, recovery studies were carried out at 80, 100 and 120% level of label claim. Inter-day and intra-day precision of the assay was determined by analyzing the drug sample at two different concentrations and % RSD were found to be less than 0.14 and 0.16 for DRO and PAR, respectively. LOD values were found to be 0.12 μg/ml, 0.15 μg/ml of DRO and 0.18 μg/ml, 0.20 μg/ml of PRA for method I and II, respectively. LOQ values were found to be 0.32 μg/ml, 0.40 μg/ml of DRO and 0.51 μg/ml, 0.58 μg/ml of PAR for method I and II, respectively.

**TABLE 1:** RESULTS OF ANALYSIS OF TABLET FORMULATION

Method	Tablet Brand	Label claim (mg/tablet)		% Label claim found*		± Standard deviation		Standard Error	
					
		DRO	PAR	DRO	PAR	DRO	PAR	DRO	PAR
I	T1	80	500	99.90	99.88	0.20	0.17	0.08	0.19
	T2	80	500	99.45	100.04	0.53	0.33	0.22	0.13
II	T1	80	500	99.42	99.80	0.94	0.25	0.39	0.47
	T2	80	500	100.30	99.94	0.46	0.51	0.41	0.21

* Average of six estimations. DRO and PAR denotes drotaverine hydrochloride and paracetamol respectively. Method I and II denote Q analysis and first order derivative method respectively. T1 and T2 are two different brands of tablet (T1- Dropar and T2- Drotin plus).

The proposed Q analysis method is also a simple and easy method. This method requires determination of absorbances of sample solution at the two selected wavelengths and few simple calculations. The first derivative spectrophotometric method requires spectral data processing and hence can be applied only on recording spectrophotometers with such facilities. This method was employed totally to eliminate the spectral interference from one of two drugs while eliminating the other drug. This was achieved by selecting the zero crossing point on the derivative spectra of one drug as the wavelength for estimation of other drug. First derivative method is simple, less time consuming, no manual calculation is required and gives better results.

Both the developed methods were found to be simple, rapid, accurate and precise for routine simultaneous estimation of both drugs in tablet dosage form. The value of standard deviation was satisfactorily low and the recovery was close to 100% indicating the reproducibility and accuracy of the methods.
